# Predictive impact of absolute lymphocyte counts for progression-free survival in human epidermal growth factor receptor 2-positive advanced breast cancer treated with pertuzumab and trastuzumab plus eribulin or nab-paclitaxel

**DOI:** 10.1186/s12885-018-4888-2

**Published:** 2018-10-16

**Authors:** Kazuhiro Araki, Yoshinori Ito, Ippei Fukada, Kokoro Kobayashi, Yoshimasa Miyagawa, Michiko Imamura, Ayako Kira, Yuichi Takatsuka, Chiyomi Egawa, Hirofumi Suwa, Shinji Ohno, Yasuo Miyoshi

**Affiliations:** 1 Hyogo College of Medicine, Department of Surgery, Division of Breast and Endocrine Surgery, 1-1 Mukogawa, Nishinomiya, Hyogo 663-8501 Japan; 2 The Cancer Institute Hospital of the Japanese Foundation for Cancer Research, Breast Medical Oncology, 3-8-31 Ariake, Koto-ku, Tokyo, 135-8550 Japan; 3 Kansai Rosai Hospital, Department of Surgery, 3-1-69 Inabaso, Amagasaki, Hyogo 660-8511 Japan; 4 Hyogo Prefectural Amagasaki General Medical Center, Department of Breast Surgery, 2-17-77 East Namba-machi, Amagasaki, Hyogo 660-8550 Japan

**Keywords:** Absolute lymphocyte counts, Predictive biomarker, Pertuzumab, Trastuzumab, HER2-positive ABC, Host immunity

## Abstract

**Background:**

Although peripheral blood-based parameters (PBBPs) are reported as prognostic indicators in patients with breast cancers, their utility has not been studied in human epidermal growth factor receptor 2 (HER2)-positive advanced breast cancer (ABC). Tumor-infiltrating lymphocytes (TILs) might be a predictive factor in patients with HER2-positive ABC treated with pertuzumab and trastuzumab (PT) plus docetaxel. We aimed to evaluate whether PBBPs could have predictive value in HER2-positive ABC treated with pertuzumab and trastuzumab (PT) combined with eribulin (ERI) or nab-paclitaxel (Nab-PTX).

**Methods:**

Data from 51 patients included in two single-arm, phase II trials were included in this retrospective-prospective study; the ERI + PT (*N* = 30) and Nab-PTX + PT (*N* = 21) combinations were registered under clinical trials number UMIN000012375 and UMIN000006838, respectively. We assessed PBBPs using prospectively collected data and investigated the association with progression-free survival (PFS); we evaluated absolute lymphocyte count (ALC), neutrophil to lymphocyte ratio (NLR) and platelet to lymphocyte ratio (PLR). The cutoff values for ALC, NLR, and PLR were set at 1000 or 1500 cells/μL, 2, and 250, respectively.

**Results:**

PFS was significantly improved in patients with ALC ≥1500/μL compared to those with ALC 1000–, <1500/μL or ALC < 1000/μL (*P* = 0.0106). High baseline ALC was significantly associated with improved PFS (≥1500/μL; hazard ratio [HR]: 0.3715; 95% confidence interval [CI]: 0.1735–0.7955; *P* = 0.0108). In contrast, improved PFS was not significantly associated with NLR or PLR. Improved PFS in patients with ALC ≥1500/μL was observed irrespective of visceral metastasis or chemotherapy regimen.

**Conclusions:**

Our results showed that baseline ALC was a predictive factor for PFS in HER2-positive ABC treated with PT irrespective of combined chemotherapy regimen. Anti-tumor effects might be mediated not only by the tumor microenvironment, but also by systemic peripheral circulating lymphocytes. Baseline systemic parameters such as peripheral lymphocyte count might be beneficial in addition to disease extent for predicting the efficacy of PT treatment.

**Trial registration:**

UMIN000012375, registration date: 21NOV2013, and UMIN000006838, registration date: 6DEC2011.

## Background

During the past decade, advanced human epidermal growth factor receptor 2 (HER2)-targeted therapies have significantly improved outcomes for patients with HER2-positive advanced breast cancer (ABC) [1]. Clinical evaluations of pertuzumab and trastuzumab (PT) in combination with different chemotherapy regimens in the CLEOPATRA trial and our previous study have highlighted clinical benefits [2, 3].

Pertuzumab and trastuzumab are humanized monoclonal anti-HER2 antibodies with complementary proposed mechanisms of action. Both antibodies work through the disruption of HER2 dimerization with HER3 and other epidermal growth factor receptors (EGFRs), leading to the inhibition of HER2 signaling [4]. Anti-HER2 antibodies are thought to mediate tumor regression not only by interrupting oncogenic signaling, but also by inducing antibody-dependent cell-mediated cytotoxicity (ADCC) [4]. ADCC can induce innate immune system responses via Fcγ receptors. These responses are sustained by natural killer (NK) cells; concomitantly, adaptive immunity involving tumor-infiltrating lymphocytes (TILs) develops in response to a specific antigen, which is sustained by T and B lymphocytes [5]. The association of TILs with clinical outcomes suggests a significant role for antitumor immunity in HER2-positive ABC [6].

Immune responses against cancer cells might involve a balance between tumor inhibitory mechanisms induced by intrinsic immunity and the ability of tumor cells to evade immune surveillance [7]. During cancer immunoediting phases (i.e., elimination, equilibrium, and escape), activated effector cytotoxic T lymphocytes can migrate to the tumor and infiltrate the tumor microenvironment [7, 8]. It has been reported that circulating peripheral lymphocytes and neutrophils can migrate towards a tumor site in a directed manner along the humoral factors, such as a chemoattractant [9]. A recent study demonstrated that circulating tumor-specific T lymphocytes related to neoantigens can be isolated from the peripheral blood of patients with melanoma [10]. These data may indicate that blood-based parameters (PBBPs) reflect a local immune reaction against cancer cells. In line with such a mechanism, the absolute lymphocyte count (ALC), neutrophil-to-lymphocyte ratio (NLR), and platelet-to-lymphocyte ratio (PLR) are reported to be immune-related prognostic factors for patients with malignancy [11, 12]. In addition, relationships between low levels of NLR and improved response to neoadjuvant chemotherapy have been reported for patients with breast cancer [13–15]. Therefore, PBBPs seem to affect treatment efficacy for ABC treated with chemotherapy and anti-HER2 therapy; however, this topic has yet to be fully studied.

In the present study, we aimed to explore whether PBBPs are related to improved progression-free survival (PFS) in patients with HER2-positive ABC treated with HER2-targeted PT combined with eribulin (ERI) or nab-paclitaxel (Nab-PTX).

## Methods

### Study design and participants

#### Patients

Data from 51 patients included in two single-arm, phase 2 trials were included in this retrospective–prospective study (ERI + TP [*N* = 30] and Nab-PTX + TP [*N* = 21]) registered under University Hospital Medical Information Network (UMIN)000012375 and UMIN000006838, respectively, and approved by the ethics committee of The Cancer Institute Hospital of the Japanese Foundation for Cancer Research (#2013–1096 and #2017–1076) and the Hyogo College of Medicine (No. 1061 and No. 2720), respectively.

### Drug dosing schedule in two prospective phase II studies

In the UMIN000006838 study, Nab-PTX 260 mg/m^2^ was administered intravenously over 30 min every 3 weeks. Two-step dose reductions to 220 mg/m^2^ and 180 mg/m^2^ were allowed before discontinuation was considered. Details of the dosing schedule in the UMIN000012375 study were published in our previous report [3]. Dose reductions were allowed for both drugs but not for PT, whereas dose interruptions were allowed for all drugs. PT was administered according to a standard procedure as published previously [3].

### Evaluation of predictive factors

Data for PBBPs at baseline were obtained on the same day and just before the start of the first cycle of chemotherapy administration. No patient was administered granulocyte-colony stimulating factor at the last cycle of prior chemotherapy. Based on our study protocol, registration criteria required an interval of more than 3 weeks since a patient’s last chemotherapy to minimize the influence of prior cytotoxic treatment. We evaluated ALC, NLR, and PLR data at baseline; the cutoff values for ALC, NLR, and PLR were set at 1000 or 1500 cells/μL, 2, and 150, respectively. Cutoff values for both NLR and PLR were defined as the median value of each parameter. The cutoff value for ALC was based on the previous study [16].

### Statistics

#### PFS analysis

PFS was defined as the time from treatment initiation to the date of disease progression or death; it was censored at the last adequate tumor assessment date before the cutoff date if no PFS event was observed prior to that time. PFS distributions were estimated using the Kaplan-Meier method.

#### Univariate and multivariate analyses

To evaluate the relationship between PFS and all clinical factors, univariate Cox proportional hazard regression models were used. Candidate predictive factors with a *P*-value less than 0.05 by univariate analysis were selected for evaluation by multivariate analysis. The value of ALC in terms of PFS was evaluated using a forest plot for each subgroup, dichotomized as < 1500 and ≥ 1500 cells/μL.

We tested associations between ALC and baseline clinicopathological characteristics using the Wilcoxon rank sum test for continuous variables with two groups for age and previous chemotherapy regimens, and Fisher’s exact test for other categorical variables, as shown in Tables 1 and 3. All statistical analyses were performed using JMP Pro 13 (SAS Institute Inc., Cary, NC, USA) and GraphPad Prism 6 (GraphPad Software, Inc., La Jolla, CA, USA). *P* values of less than 0.05 were considered statistically significant.

**Table 1:** Demographics and baseline characteristics of patients

		Eribulin (*N* = 30)	Nab-paclitaxel (*N* = 21)	Total (*N* = 51)	*P* value
Median age	(range)	58 (31–76) yo	58 (32–77) yo	58 (31–77)	0.5524
Performance status	0	10 (33%)	20 (95%)	30 (59%)	< 0.0001
	1	18 (60%)	1 (5%)	19 (37%)	
	2	2 (7%)	0	2 (4%)	
HER2 immunohistochemical staining	3+	25 (83%)	15 (71%)	40 (78%)	0.327
	2+ (with FISH amplification)	5 (17%)	6 (19%)	11 (22%)	
Estrogen receptor	positive	14 (47%)	9 (43%)	23 (45%)	1.000
	negative	16 (53%)	12 (57%)	28 (55%)	
Progesterone receptor	positive	5 (17%)	6 (19%)	11 (22%)	0.327
	negative	25 (83%)	15 (71%)	40 (78%)	
Metastatic sites	visceral	15 (50%)	4 (19%)	19 (37%)	0.0389
	non-visceral	15 (50%)	17 (81%)	32 (63%)	
Previous chemotherapy regimens, median	(range)	3.5 (1–9)	1 (1–10)	3 (1–10)	0.0579

## Results

### Demographics and baseline clinical characteristics

Table 1 shows a summary of the baseline clinical characteristics of patients from both studies and the differences between the ERI and Nab-PTX groups. A total of 51 patients (median age, 58 years) were enrolled; of these, 49 patients (96%) had favorable Eastern Cooperative Oncology Group Performance Status (ECOG-PS) scores of 0 or 1. Patients in the Nab-PTX group had significantly better ECOG-PS than those in the ERI group (*P* < 0.0001). Forty patients (78%) had HER2 overexpression (3+) confirmed by immunohistochemistry (IHC), and 11 patients (22%) with IHC 2+ status were confirmed to be HER2 positive by fluorescence in situ hybridization. Twenty-three cases (45%) were estrogen receptor (ER) positive, whereas 11 patients (22%) were progesterone receptor (PgR) positive. Visceral metastases and non-visceral metastases were reported in 19 (37%) and 32 (63%) patients, respectively. Patients with visceral metastases (50%) were significantly more in the ERI group than in the Nab-PTX group (19%, *P* = 0.0389). Patients received a median of 3 (range, 1–10) prior treatments. There was no significant difference between groups in any parameters other than PS and metastatic sites.

### Efficacy

#### Anti-tumor activity

All patients in the full-analysis set were evaluable for overall response rate (ORR). Investigator assessments identified 4 patients with complete response (8%), while 15 patients (29%) achieved partial response. The ORR was 37%, the disease control rate was 78% (*N* = 40), and the clinical benefit rate was 55% (*N* = 28). The median PFS was 301 days (95% confidence interval [CI]: 212–568) based on Kaplan–Meier estimates. There was no significant difference in PFS between ERI and Nab-PTX (hazard ratio [HR]: 1.593, 95% CI [0.7510–3.378], *P* = 0.2249).

#### Univariate and multivariate analyses of PFS according to the baseline clinical factors

Table 2 provides a summary of univariate and multivariate analyses of PFS according to baseline clinical characteristics. Univariate analysis showed that patients aged < 58 years, those with ALC ≥1000/μL and ALC ≥1500/μL, and those with less than 3 lines of prior treatment had better PFS (Table 2). Both NLR and PLR showed a trend toward statistical significance (Table 2). ER and PgR positivity, HER2–3+, presence of visceral metastases, and treatment during study were not significant predictors of PFS (Table 2).

**Table 2:** Univariate and multivariate analyses of progression-free survival

	Number of patients		Univariate			Multivariate		
Parameter	Yes	No	HR	95% CIs	*P* value	HR	95% CIs	*P* value
Age < 58 yo	23/51	28/51	2.440	1.120–5.316	0.0248	0.315	0.074–1.467	0.1367
Estrogen receptor positive (vs. negative)	23/51	28/51	1.198	0.555–2.588	0.6448			
Progesterone receptor positive (vs. negative)	11/51	40/51	1.177	0.456–3.040	0.7361			
HER2 3+	40/51	11/51	1.421	0.558–3.618	0.5431			
Non-visceral metastases (vs. visceral)	32/51	19/51	0.926	0.429–1.996	0.8441			
Eribulin (vs. Nab-PTX)	30/51	21/30	1.593	0.751–3.378	0.2249			
Prior treatment < 3 line	30/51	21/30	0.401	0.185–0.870	0.0208	0.528	0.234–1.157	0.1106
Absolute lymphocyte count ≥ 1000/uL	37/51	14/51	0.308	0.133–0.714	0.0061	0.735	0.284–1.770	0.4977
Absolute lymphocyte count ≥ 1500/uL	19/51	32/51	0.372	0.174–0.796	0.0108	0.296	0.098–0.794	0.0150
Neutrophil to lymphocyte ratio > 2	31/51	20/51	2.109	0.985–4.516	0.0548			
Platelet to lymphocyte ratio > 150	31/51	20/51	2.038	0.962–4.318	0.0632			

**Fig. 1:**
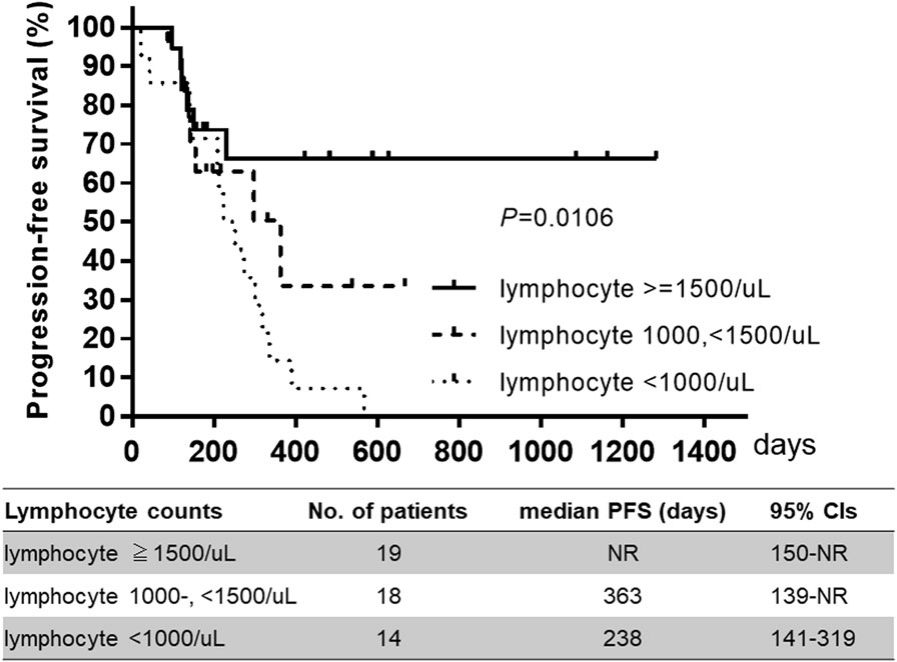
Comparison of progression-free survival in patients according to absolute lymphocyte counts. Progression-free survival (PFS) was significantly longer in patients with absolute lymphocyte counts (ALC) ≥1500/μL (*P = 0.0106*). Solid lines indicate ALC ≥1500/μL, broken lines indicate ALC 1000–, < 1500/μL, and dotted lines indicate ALC < 1000/μL. Time (days) indicates the duration from the start of treatment to the occurrence of events. NR, not reached

**Fig. 2:**
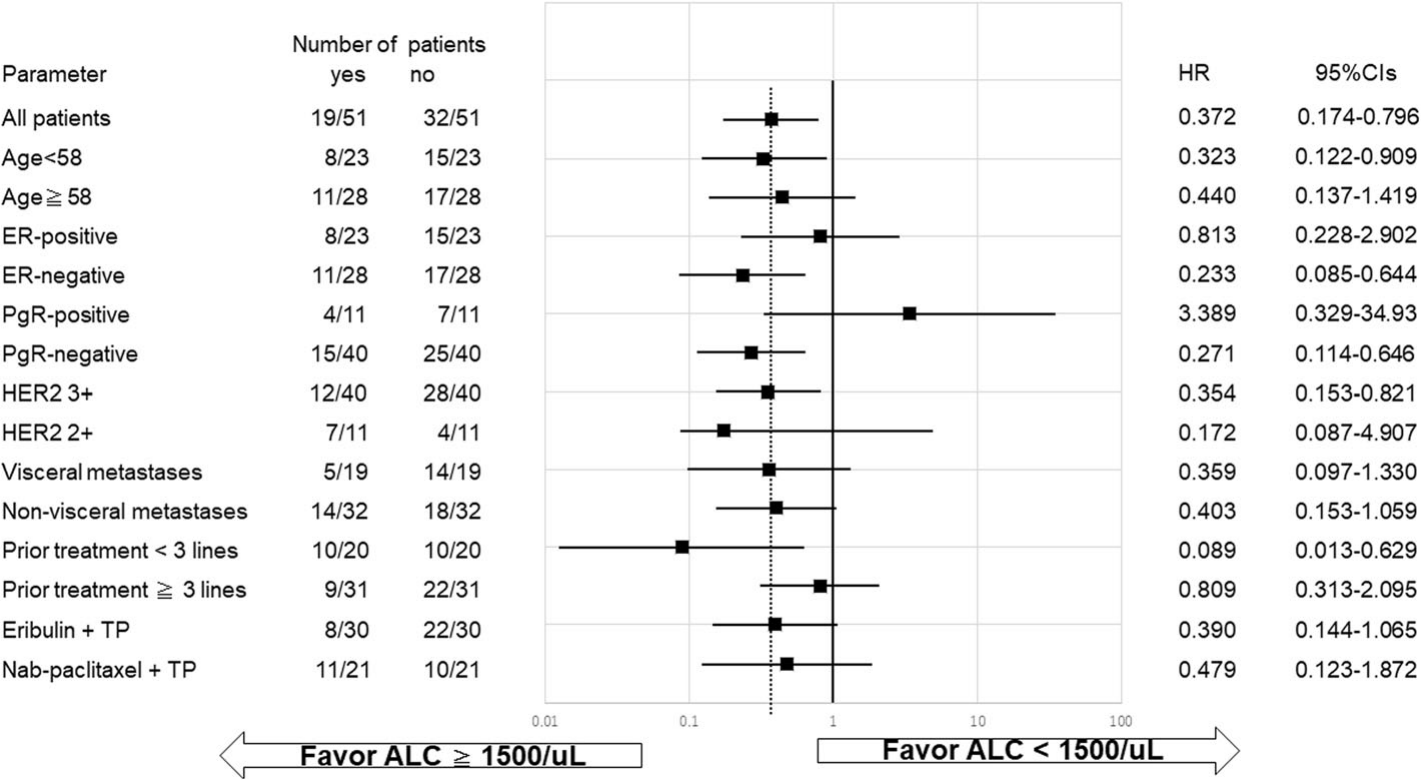
Forest plot showing hazard ratios for progression-free survival. The forest plots indicate the hazard ratios (HRs) and 95% confidence intervals (CIs) according to each factor. Evaluation of factors contributing to the prolongation of progression-free survival (PFS) with baseline clinical factors, stratified with absolute lymphocyte counts (ALC) ≥1500/μL

In the multivariate analysis, only ALC ≥1500/μL (HR: 0.296; 95% CI, 0.098–0794; *P* = 0.0150) was found to be correlated with PFS (Table 2). PFS was also significantly longer in patients with ALC ≥1500/μL compared with 1000–1500/μL or < 1000/μL (median PFS: not reached versus 363 versus 238 days, respectively; *P* = 0.0106; Fig. 1). An exploratory analysis (Fig. 2) of the HRs associated with ALC ≥1500/μL or <  1500/μL consistently favored PFS in patients with ALC ≥1500/μL, irrespective of age (< 58 and ≥ 58 years), HER2 levels (3+ and 2+), metastatic sites (visceral or non-visceral), and treatment (ERI or Nab-PTX), except in cases that were PgR-positive.

A comparison of baseline characteristics based on ALC ≥1500/μL is shown in Table 3. There was no significant difference in the relationship between ALC levels and any factors, including metastatic site, number of prior treatments, or regimens.

**Table 3:** Comparison of baseline patients’ characteristics based on patients with baseline absolute lymphocyte counts

Characteristics	Absolute lymphocyte counts		*P* value
	≥ 1500/μL (*n* = 19)	< 1500/μL (*n* = 32)	
Median age (range)	58 (32–76) yo	58 (31–77) yo	0.7109
Estrogen receptor			
Positive	8 (42%)	15 (47%)	0.7787
Negative	11 (58%)	17 (53%)	
Progesterone receptor			
Positive	4 (21%)	7 (22%)	1.0000
Negative	15 (79%)	25 (78%)	
HER2			
3+	12 (63%)	28 (88%)	0.0754
2+ FISH amplification	7 (37%)	4 (12%)	
Visceral metastases	5 (26%)	14 (47%)	0.2465
Non-visceral metastases	14 (74%)	18 (56%)	
Prior treatment			
< 3 lines	13 (68%)	17 (53%)	0.3808
≥ 3 lines	6 (32%)	15 (47%)	
Combination chemotherapy with trastuzumab plus pertuzumab			
Eribulin	8 (42%)	22 (69%)	0.0815
Nab-paclitaxel	11 (58%)	10 (31%)	

## Discussion

In this prospective-retrospective evaluation of PBBPs in baseline clinical data, we identified ALC as a predictive factor for PFS in patients with HER2-positive ABC. Additionally, high ALC at baseline was significantly associated with improved PFS in HER2-positive ABC treated with either ERI or Nab-PTX in combination with PT. To the best of our knowledge, this study is the first to analyze predictive factors associated with ALC in HER2-positive ABC. For treatment of ABC, predictive factors related to systemic immune response are yet to be considered in clinical practice.

Usually, somatic mutations in cancer cells lead to the production of altered proteins that are recognized as antigens by the innate immune system via major histocompatibility complex class I; consequently, TILs inhibit tumor progression [17]. Anti-HER2 antibodies also mediate anticancer effects in part via the induction of ADCC by opsonizing cancer cells that are recognized by the innate immune system [18]. Anti-HER2 antibodies not only neutralize the trophic function of HER2, but they also elicit an initial NK-mediated ADCC response that is presumably followed by a cytotoxic T lymphocyte-dependent adaptive immune repose directed against breast cancer associated antigens [18]. The effect of pertuzumab in antitumor immunity is still unknown. In a subgroup analysis of the CLEOPATRA trial, patients with a high abundance of TILs (> 20%) had better PFS than those with low TILs (≤20%) in the PT group, but not in the trastuzumab group [6]. These data might indicate that preexisting immune responses enhance treatment efficacy, which could be boosted by combination therapy with conventional chemotherapy and PT [19]. Although prognostic markers including NLR and PLR have been evaluated in several malignant diseases [12], our study demonstrated that ALC is superior to NLR and PLR for predicting improved PFS in ABC patients treated with conventional chemotherapy combined with PT. High ALC may indicate enhanced immunity in tumors; alternatively, ALC may be a potential biomarker of host immunity. Although the detailed underlying mechanism of prolonged PFS in patients with ALC ≥1500/μL is currently unknown, it is likely that these results reflect the synergistic activity of PT acting alongside the host immune response.

Our results showed that the favorable PFS in patients with ALC ≥1500/μL was prominent in the ER- and PgR- subgroups. It is well established that TILs are not frequently observed in ER+/HER2- breast cancers [20]; thus, the prognostic significance of TILs is not recognized in these cases [20]. Even among HER2+ breast cancers, the frequency of TILs was significantly lower in ER+ cases compared with ER- cases [6]. These data indicate that anticancer immunity is not dominant in ER+ cases. Thus, ALC may be a less effective predictor in ER+ and PgR+ cases compared to ER- and PgR- cases.

Both ERI and Nab-PTX could be classified as microtubule inhibitors with differential mechanisms of action [21]. Both drugs can cause G2/M cell cycle arrest due to disruption of mitotic spindles, resulting in apoptotic cell death after prolonged mitotic blockage [22]. In the current study, a favorable PFS in patients with ALC ≥1500/μL was observed, irrespective of a combination treatment with ERI or Nab-PTX. Because chemotherapies differ with regard to immunogenicity (e.g., immunogenic [doxorubicin or cyclophosphamide] or non-immunogenic [dacarbazine] [21]), it is unclear whether relationships exist between PFS and ALC in patients treated with PT in combination with other chemotherapies or endocrine therapies.

This study has several limitations. First, we used a retrospective design from two single prospective phase 2 studies with relatively small sample sizes. Second, prior chemotherapies may affect baseline levels of PBBPs. However, because a positive association between ALC levels and PFS was consistently recognized in patients with and without prior chemotherapy, the influence of prior chemotherapy appears to be limited. There was no significant difference for any PBBP during the course of treatment (data not shown), but the prognostic significance of PBBPs during the treatment course is currently unknown. Considering these limitations, prospective studies that include a large number of patients are needed to further elucidate the results of the present study.

## Conclusions

Our findings strongly suggest that ALC at baseline is a useful predictor for prolonged PFS in HER2-positive ABC patients treated with PT in combination with ERI or Nab-PTX. Baseline ALC may be indicative of pre-existing anti-tumor host immunity or the potential immune response following PT-based therapy. Future research might focus on the baseline systemic immune response evaluable by ALC in daily clinical practice.

## Abbreviations

ABC: Advanced breast cancer
ADCC: Antibody-dependent cell-mediated cytotoxicity
ALC: Absolute lymphocyte count
CI: Confidence interval
CLEOPATRA: The investigators in the Clinical Evaluation of Pertuzumab and Trastuzumab
ECOG-PS: Eastern Cooperative Oncology Group Performance Status
EGFR: Epidermal growth factor receptor
ER: Estrogen receptor
ERI: Eribulin
HER2: Human epidermal growth factor receptor 2
HR: Hazard ratio
IHC: Immunohistochemistry
JSPS: Japan Society for Promotion of Science
Nab-PTX: Nab-paclitaxel
NL: Natural killer
NLR: Neutrophil to lymphocyte ratio
PBBPs: Peripheral blood-based parameters
PFS: Progression-free survival
PgR: Progesterone receptor
PLR: Platelet to lymphocyte ratio
PT: Pertuzumab and trastuzumab
TILs: Tumor-infiltrating lymphocytes
